# Pertussis Vaccines Scarcely Provide Protection against *Bordetella parapertussis* Infection in Children—A Systematic Review and Meta-Analysis

**DOI:** 10.3390/vaccines12030253

**Published:** 2024-02-28

**Authors:** Arun Thachappully Remesh, Kalichamy Alagarasu, Santoshkumar Jadhav, Meera Prabhakar, Rajlakshmi Viswanathan

**Affiliations:** 1 Bacteriology Group, ICMR-National Institute of Virology, Pune 411021, India; arun.tr@icmr.gov.in (A.T.R.); meera6396@gmail.com (M.P.); 2 Dengue-Chikungunya Group, ICMR-National Institute of Virology, Pune 411001, India; alagarasu.k@gov.in; 3 Bioinformatics & Data Management Group, ICMR-National Institute of Virology, Pune 411001, India; jadhav.sm@gov.in

**Keywords:** pertussis, parapertussis, whole cellular pertussis vaccine, acellular pertussis vaccine, protection, effectiveness

## Abstract

Background: Pertussis, or whooping cough, is a global public health concern. Pertussis vaccines have demonstrated good protection against *Bordetella pertussis* infections, but their effectiveness against *Bordetella parapertussis* remains debated due to conflicting study outcomes. Methods: A systematic review and meta-analysis were conducted to assess the effectiveness of pertussis vaccines in protecting children against *B. parapertussis* infection. A comprehensive search of PubMed, Web of Science, and Scopus databases was conducted, and randomized controlled trials (RCTs) and observational studies that met inclusion criteria were included in the analysis. Results: The meta-analysis, involving 46,533 participants, revealed no significant protective effect of pertussis vaccination against *B. parapertussis* infection (risk ratio: 1.10, 95% confidence interval: 0.83 to 1.44). Subgroup analyses by vaccine type and study design revealed no significant protection. The dearth of recent data and a limited pool of eligible studies, particularly RCTs, underscore a critical gap that warrants future research in the domain. Conclusions: These findings offer crucial insights into the lack of effectiveness of pertussis vaccines against *B. parapertussis*. Given the rising incidence of cases and outbreaks, coupled with the lack of cross-protection by the existing vaccines, there is an urgent need to develop vaccines that include specific antigens to protect against *B. parapertussis*.

## 1. Introduction

*Bordetella pertussis* is a well-recognized and documented etiology of pertussis, or whooping cough, a very contagious respiratory infection [1]. *B. parapertussis* causes a similar albeit often milder illness that cannot be clinically distinguished from *B. pertussis* and is usually not laboratory-confirmed [2]. The identification of *B. parapertussis* dates back to 1937, when an organism biochemically dissimilar to *B. pertussis* was isolated from pediatric cases of whooping cough by two intrepid female scientists, Eldering and Kendrick [3]. Later named *Bordetella parapertussis*, this organism has long been studied less than *B. pertussis.* Recently, *B. parapertussis* has become increasingly prominent, with a growing prevalence and potential contribution to the overall pertussis burden [4,5]. Outbreaks of *B. parapertussis*-associated whooping cough have been reported globally [6,7,8].

*B. pertussis* and *B. parapertussis* are closely related and, together with *B. bronchiseptica,* make up the ‘classic’ or ‘mammalian’ bordetellae [9]. Based on the evaluation of genomic sequences, both *B. pertussis* and *B. parapertussis* are considered to have evolved independently from a common *B. bronchispectica-like* ancestor [9]. Both the species have similar common virulence factors, such as pertactin (PRN), filamentous hemagglutinin (FHA), adenylate cyclase toxin, and heat-labile toxin, with the notable exception of pertussis toxin, which is specific to *B. pertussis* [10].

Vaccination for pertussis came into effect almost 75 years ago, initially with the heat-killed whole cellular pertussis vaccine (wP) and later the acellular vaccine (aP), which includes several purified antigens of *B. pertussis* [11]. However, the effectiveness of pertussis vaccines against *B. parapertussis* remains uncertain. Studies have provided contradictory results regarding the protective effect of pertussis vaccines against *B. parapertussis*. Stehr et al. found the aP vaccine to be effective (31%) as compared to 6% for the wP vaccine, albeit with a high margin of error [12]. On the contrary, some investigations found that the aP vaccines had little efficacy against *B. parapertussis* [13]. The effectiveness of the pertussis vaccine in preventing *B. parapertussis* infections among children in Oregon was calculated using two different methods (relative risk and indirect cohort method). The evaluations revealed vaccine effectiveness of 66% and 82%, respectively, indicating that the pertussis vaccine may potentially induce cross-immunity against *B. parapertussis* [14].

Animal studies also provide conflicting evidence. Some studies reported a significant protective effect of wP vaccines against *B. parapertussis* [15,16]. Reciprocal protection was induced in mice infected with *B. pertussis* and those infected with *B*. *parapertussis* [17]. It was postulated that Th1 and Th2 responses against FHA might have a role in mediating the reciprocal protection observed. However, some investigations found that the aP vaccines were ineffective against *B. parapertussis* [18]. PRN, an outer membrane protein, is known to induce bactericidal antibodies that mediate bacterial clearance [19]. PRN of *B. pertussis* and *B. parapertussis* are known to have different immunogenic properties. Studies have shown that PRN preparations of *B. pertussis* protect mice against intranasal challenge with the same species, but not against a similar challenge with *B. parapertussis* [10]. However, a recent study in mice, of recombinant antibodies binding four distinct epitopes on PRN, demonstrated that four of these antibodies bind epitopes across *B. pertussis* and *B. parapertussis,* as well as their common ancestor, *B. bronchiseptica* [20].

Considering the diverse range of existing evidence, this systematic review and meta-analysis is designed to collate and synthesize the current information regarding the efficacy of pertussis vaccines in preventing *B. parapertussis* infection among children, who represent the most frequently affected age group.

## 2. Materials and Methods

### 2.1. Search Strategy

The study protocol was registered on PROSPERO (CRD42023453005 date 21 August 2023). The recommendations of the Preferred Reporting Items for Systematic Reviews and Meta-Analyses (PRISMA) guidelines were followed for reporting this systematic review [21].

Three databases: MEDLINE, Web of Science, and Scopus, were searched from 1948 to 31 March 2023, using the search terms ((((pertussis) OR (“whooping cough”)) OR (“*Bordetella pertussis*”)) AND ((vaccin*) OR (immun*))) AND (parapertussis).

### 2.2. Eligibility Criteria

The inclusion criteria were (1) randomized controlled trials (RCTs) or observational studies (cross-sectional, case-control, or cohort study designs) with (2) participants <19 years, (3) reporting laboratory-confirmed *B. parapertussis* infections, and (4) pertussis vaccination data.

Exclusion criteria were studies involving (1) participants >19 years of age, (2) in vitro and animal studies, (3) studies that did not report laboratory-confirmed *B. parapertussis* infection and/or pertussis vaccination status, (4) studies in which all participants were either vaccinated or unvaccinated. Case reports, editorials, opinion pieces, letters, and review articles were not included.

The selection criteria (PICO) were as follows:

Population (P): children less than 19 years of age.

Intervention (I): vaccination with either whole-cellular or acellular pertussis vaccines.

Comparison (C): unvaccinated individuals or vaccinated with formulations without pertussis antigens (e.g., DT vaccine).

Outcome (O): incidence of laboratory-confirmed *B. parapertussis* infections

The selection criteria, based on the PICO framework, were defined as follows: population (P) included children less than 19 years of age; Intervention (I) entailed vaccination with either whole-cell or acellular pertussis vaccines; Comparison (C) was made with unvaccinated individuals or those vaccinated with formulations lacking pertussis antigens, such as the DT vaccine; and the Outcome (O) focused on the incidence of laboratory-confirmed *B. parapertussis* infections.

### 2.3. Selection of Studies

The articles retrieved from all three databases (PubMed, Scopus, and Web of Science) were imported into RAYYAN (https://www.rayyan.ai/) [22]. Duplicate articles were removed using the RAYYAN software. Two reviewers (ATR and MP) independently screened the titles and abstracts of the retrieved articles against the established inclusion and exclusion criteria to identify potentially relevant studies. Articles that seemed to meet the inclusion criteria, or those with uncertain eligibility based on the title and abstract screening, underwent a full-text review. Two reviewers (ATR and MP) independently conducted this full-text review of the potentially relevant studies. Any discrepancies between the two reviewers were resolved by discussion and consensus following an independent assessment by a third reviewer (RV). A follow-up search employing the same search terms was conducted to retrieve studies published after the initial search.

### 2.4. Data Extraction

Two reviewers, ATR and MP, independently extracted data from the included studies, utilizing a predefined form designed for data extraction [Table S1]. This process involved gathering detailed study characteristics, including the publication title, author names, year of publication, country, study period, study design, and the diagnostic test used for the outcome measurement, as well as the total participant count. Additionally, they collected data on study results, such as the number of *B. parapertussis* cases, the count of vaccinated and unvaccinated individuals who tested both positive and negative for *B. parapertussis*, instances of partial vaccinations, if any, and the number of participants with unavailable vaccination data. Moreover, information regarding the type of intervention (vaccine) and the type of the comparison group was also extracted. Any discrepancies or disagreements between the two reviewers in the data extraction process were resolved through discussion and consensus or, if needed, by consulting a third reviewer.

### 2.5. Quality Assessment

The Cochrane Risk of Bias tool (RoB2) was employed for evaluating RCTs [23], and the Newcastle-Ottawa Quality Assessment Scale (NOS) was used for case-control and cohort studies [24]. The RoB2 tool covers five domains with 22 questions addressing different sources of bias. The domains focus on bias arising from the randomization process, deviations from intended interventions, missing outcome data, and measurements of the outcome, and the selection of the reported results are assessed. The studies were rated as “yes/no/partially yes/partially no” for each question, with an overall judgment of the risk of bias provided. The NOS used criteria grouped into three domains: selection, comparability, and exposure (for case-control studies) or outcome (for cohort studies). Each criterion was rated as “Yes”, “No”, or “Not Applicable”. The criteria were ranked with stars, and an overall quality assessment was made based on the total number of stars earned by the study.

### 2.6. Statistical Analysis

Data analysis was performed using STATA software version 16.1, employing the meta-analysis module. The restricted maximum likelihood (REML) method was used for estimating the between-study variance in a random-effects model. The protective effect of pertussis vaccination was described with risk ratios (RR) along with 95% confidence intervals (CI). The RR was used to compare the risk of *B. parapertussis* infection between the vaccinated and unvaccinated groups, where an RR greater than 1 indicated an increased risk among the vaccinated individuals. Vaccine effectiveness (VE) was calculated using (1 − RR) × 100% for pooled estimates of RR. Forest plots were used to display pooled estimates. The pooled effect size (θ), representing the combined risk ratio (RR) of *B. parapertussis* infection between vaccinated and unvaccinated individuals, was estimated. The θi values represented the individual study effect sizes, contributing to the overall θ. Between-study variance (τ^2^) was estimated to assess the variability in effect sizes across studies. Heterogeneity was estimated by visual inspection of forest plots. Quantification was reported by the I^2^ statistic, with I^2^ > 40% representing moderate, >60% substantial, and >80% considerable heterogeneity [25]. H^2^ provided an estimate of the total amount of heterogeneity in the meta-analysis. An H^2^ value of 1 suggests no heterogeneity, while values greater than 1 indicate the presence of heterogeneity. A continuity correction to zero cells was applied to include all studies in the analysis while minimizing bias [26]. Sub-group analysis was conducted based on the type of study design and the type of intervention, distinguishing between wP and aP vaccines.

### 2.7. Publication Bias

Publication bias was assessed using the funnel plot, with the log risk ratio on the *x*-axis and standard error on the *y*-axis [27,28].

## 3. Results

### 3.1. Literature Search and Characteristics of the Studies Included

**Figure 1:**
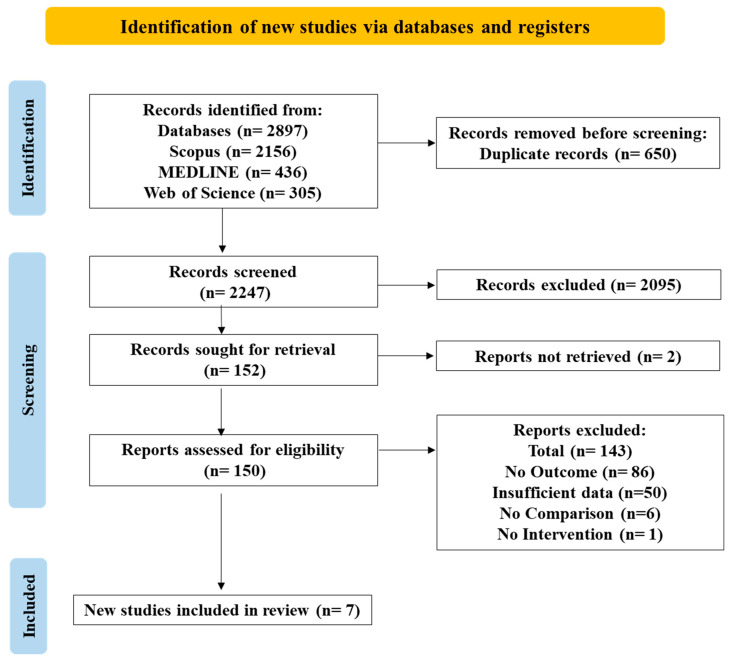
PRISMA flowchart showing identification and screening process of studies.

The search strategy initially identified 2897 articles, including 2156 from Scopus, 436 from MEDLINE, and 305 from Web of Science. A total of 650 duplicate articles were eliminated, leaving 2247 articles for title and abstract screening. Of these, 2095 articles were excluded, and 152 articles proceeded to a full-text review. Of the 150 articles retrieved and screened, 86 reported no outcome of *B. parapertussis* infection, 50 had insufficient data, six lacked a comparison group of unvaccinated individuals, and one had no intervention group of vaccinated individuals. Included among the studies with insufficient data were 18 that reported both *B. pertussis* and *B. parapertussis* infections but lacked vaccination status data. Additionally, 31 studies, including RCTs, assessed the effectiveness of the pertussis vaccine and reported cases of both infections. These studies, however, did not have separate vaccination status data for participants with *B. parapertussis* infection. One study did not possess data on negative cases of *B. parapertussis*, so that calculation of vaccine efficacy was not possible. Following the selection process, seven articles that met the inclusion criteria were chosen for analysis. The follow-up search led to the identification of ten new studies, but none met the predefined inclusion criteria. The specifics of the search process and study selection are outlined in the PRISMA flow chart (Figure 1; Table S2).

Table 1 provides a comprehensive overview of the included studies, offering details on various aspects such as the study period, participant age at enrollment, type of intervention and comparison, and the total number of participants, encompassing both those who were vaccinated and unvaccinated, and whether they contracted *B. parapertussis* infection or not. The seven articles included in the analysis consisted of four RCTs, two case-control studies, and one observational study.

### 3.2. Quality of Included Studies

The Cochrane RoB2 tool was applied to assess the risk of bias in the four RCTs [Table S3]. For SR1 by Stehr et al. [12], a low risk of bias was identified in four domains: deviations from intended interventions, missing outcome data, measurement of the outcome, and selection of the reported result. However, there was a high risk of bias in the randomization process, resulting in an overall high risk of bias for the study. Both SR2 by Mastrantonio et al. [2] and SR3 by(Bergfors et al. [29] had low bias risks in all domains except for the selection of the reported result, contributing to their overall high risk of bias. SR4 by Heininger et al. [30] exhibited a high bias risk in the randomization process, some concerns in deviations from intended interventions, and low risks in other domains, culminating in an overall high risk of bias. Despite the high overall bias risk primarily attributed to a single domain, these RCTs provided valuable data for assessing the effectiveness of pertussis vaccines against *B. parapertussis* infection.

The risk of bias in the case-control studies, SR5 by Liese et al. [31] and SR7 by Muloiwa et al. [33], was evaluated using the NOS. This scale assigned stars to various items within the selection, comparability, and exposure categories and assigned AHRQ standards based on the threshold values. Both studies were found to have good quality in the risk of bias assessment. One cohort study, SR6 by Theofiles et al. [32], was assessed for risk of bias using NOS for cohort studies. The exposed cohort was representative of the community, the non-exposed cohort was drawn from the same community, exposure was ascertained, and there was an adequate and extended follow-up with all subjects. However, the study was rated as poor quality due to limitations in the comparability of cohorts.

### 3.3. Primary Outcome: Laboratory Confirmed B. parapertussis Infection

**Figure 2:**
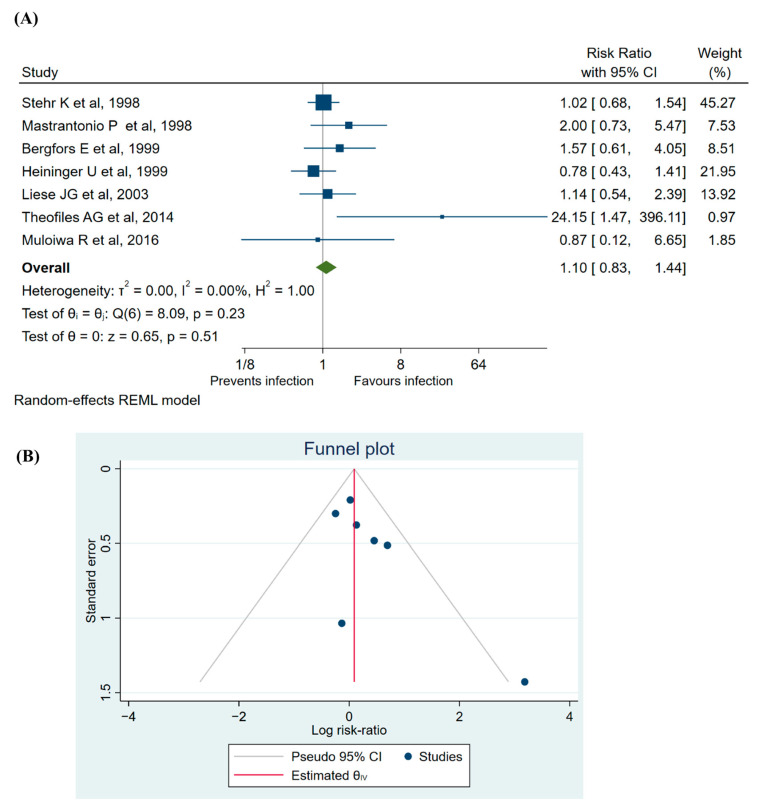
Legend. (**A**) Forest plot depicting risk ratio, that is, a risk of *B. parapertussis* infection between the vaccinated and unvaccinated groups, weight% depicts individual weightage of each study to the overall risk ratio analysis [2,12,29,30,31,32,33]. (**B**) Funnel plot showing publication bias in included articles.

The primary analysis of seven studies with 46,533 participants showed an overall risk ratio of 1.10 (95%CI 0.83 to 1.44) and VE of −10 (95%CI −44 to 17), indicating no effect of vaccination against pertussis on *B. parapertussis* infection (Figure 2A).

Heterogeneity was minimal (I^2^ = 0.00%), and the H^2^ ratio was 1.00, suggesting a consistent effect size across studies. The between-study variance (τ^2^) was effectively zero, indicating no additional variability unaccounted for by the model. Funnel plot analysis revealed no publication bias (Figure 2B). The risk ratio was significant in only one study, which contributed a mere 0.97% to the overall weightage of the analysis.

#### 3.3.1. Sub-Group Analysis by Type of Vaccination (DTP or DTaP vs. Unvaccinated and DTP vs. DTaP)

**Figure 3:**
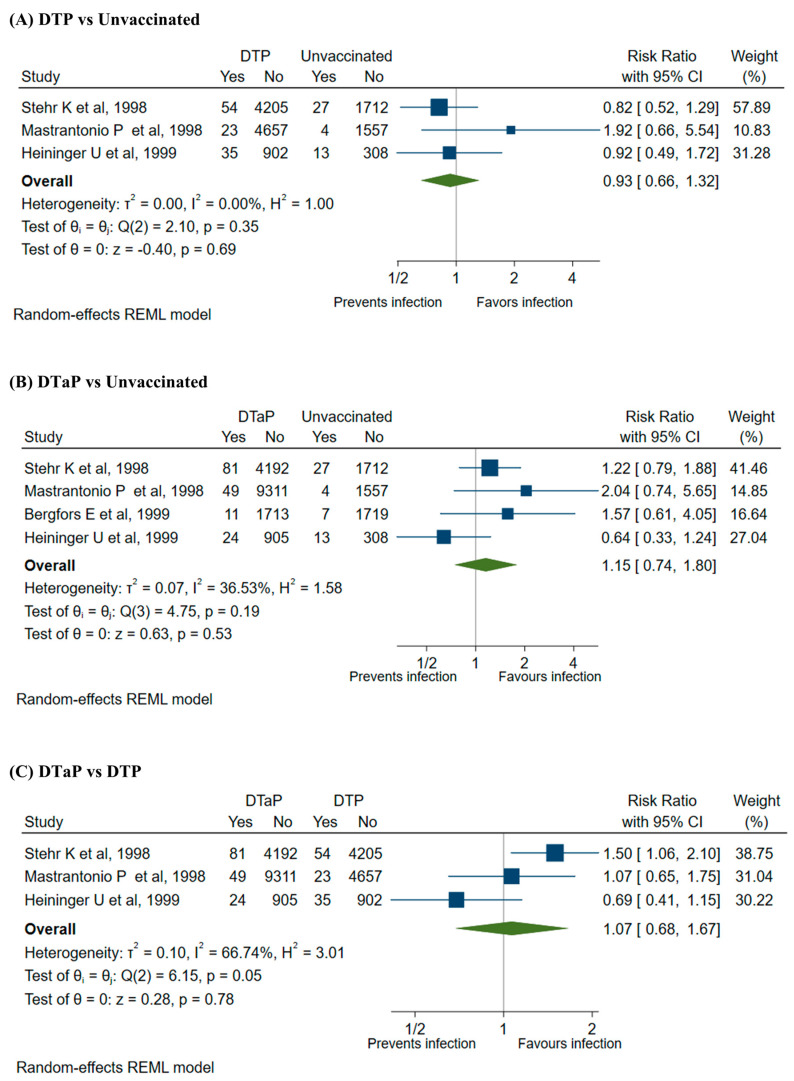
Legend. (**A**) Forest plot of risk ratio for *B. parapertussis* infection in DTP vaccinated and unvaccinated groups [2,12,30]. (**B**) Forest plot of risk ratio for *B. parapertussis* infection in DTaP vaccinated and unvaccinated groups [2,12,29,30]. (**C**) Forest plot of risk ratio for *B. parapertussis* infection in DTaP vaccinated and DTP vaccinated groups [2,12,30].

When the analysis was restricted to only those who received DTP or DTaP, no significant protective effect against *B. parapertussis* infection was observed (DTP vs. unvaccinated: RR 0.93 (95%CI 0.66–1.32), VE 7% (95%CI −32 to 34); DTaP vs. unvaccinated: RR 1.15 (95%CI 0.74–1.80) VE −15% (95%CI −80 to 26)). When the protective effect was compared between those who received DTP and those who received DTaP, no difference in the effectiveness was observed (Figure 3), and the RR and VE were 1.07 (95% CI 0.68–1.67) and 7% (95%CI −67 to 32), respectively.

#### 3.3.2. Subgroup Analysis by Study Design

**Figure 4:**
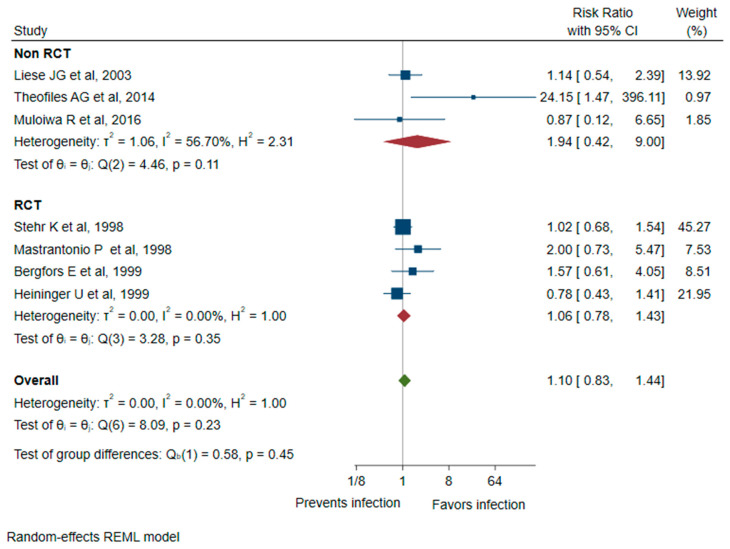
Legend. Forest plot of risk ratio for *B. parapertussis* infection between vaccinated and unvaccinated groups for both randomized controlled trials [2,12,29,30] and observational (non RCT) studies [31,32,33].

The subgroup analysis of the four RCTs, which included 31,509 participants, demonstrated a similar primary outcome (Figure 4) with an overall RR and VE of 1.06 (95%CI 0.78–1.43) and −6 (95%CI −43 to 22), respectively. No heterogeneity (I^2^ ≤ 0%) was observed. The subgroup analysis of the three other studies (two case-control and one observational) with 15,024 participants showed an RR and VE of 1.94 (95%CI 0.42 to 9.00) and −94 (95%CI −800 to 58), respectively, which was not significant.

## 4. Discussion

This systematic review and meta-analysis summarize the currently available evidence from the literature on whether vaccination against pertussis protects children against infection with *B. parapertussis*. The effectiveness of wP and aP vaccines in safeguarding against *B. pertussis* is well-established. However, *B. parapertussis* has gained recognition as an etiological agent of whooping cough worldwide [4,5,6,34,35], and its emergence has been associated with outbreaks in various regions [7,8,35]. Although *B. parapertussis* infections are typically shorter in duration than pertussis, studies have reported similar frequencies and severities of symptoms between the two pathogens [5,36,37], with the severity of *B. parapertussis* potentially greater in young infants [38].

The virulence factors produced by *B. parapertussis* and *B. pertussis* are remarkably similar, except that *B. parapertussis* lacks the pertussis toxin (PT) and the tracheal colonization factor (tcf) of *B. pertussis* [39]. Whole-cell pertussis (wP) vaccines contain inactivated *B. pertussis* organisms. The acellular pertussis (aP) vaccines comprise major antigens such as PT and other components such as filamentous hemagglutinin (FHA), fimbrial antigens (FIM2, FIM3), and pertactin (PRN) [39]. Certain vaccines have shown protective efficacy against genetically related pathogens, as exemplified by BCG vaccination, which protects against *Mycobacterium leprae* and non-tuberculous mycobacteria [40]. The potential for pertussis vaccines to confer cross-protection against *B. parapertussis* remains debated. Some studies have suggested such cross-protection, while others have reported limited or no evidence following pertussis vaccination [10,13,18,41]. Thus, it was worth exploring whether these vaccines have protective effectiveness against infection with *B. parapertussis*.

Our systematic review and meta-analysis showed no significant effect of pertussis vaccination against infection with *B. parapertussis*. Further subgroup analysis by type of study and type of vaccine also did not reveal any significant effect. No heterogeneity or publication bias was detected, although the small number of studies included may limit the ability of these tests to identify such issues. This study’s strength lies in its systematic and comprehensive methodology, adhering to the Preferred Reporting Items for Systematic Reviews and Meta-Analyses (PRISMA) guidelines to ensure transparency and rigor in the review process. Clearly defined inclusion criteria specify the types of studies, participants, and outcomes under investigation.

Nonetheless, our study encountered certain limitations primarily linked to the available literature. The exclusion of “grey literature” and reliance on peer-reviewed publications may have led to the omission of valuable data from unpublished sources. Our systematic review identified only seven eligible articles for inclusion in the analysis, whereas 50 studies presented insufficient data regarding the vaccination status of individuals with *B. parapertussis* infections [5,42,43,44,45,46]. Most studies documenting *B. parapertussis* infections focused on the efficacy of the pertussis vaccine against *B. pertussis* but overlooked the data necessary for calculating efficacy against *B. parapertussis* infections. The limited number of eligible studies, particularly RCTs, highlights the need for more comprehensive research in this domain. All included RCTs were conducted during the 1990s and early 2000s, and recent data with complete information were lacking, underscoring the need for further research. While a substantial body of literature has previously posited the hypothesis regarding the limited effectiveness of pertussis vaccines against *B. parapertussis* [6,13,18,31,41], some have suggested potential protection [12,14,15,16]. Our present investigation, anchored in the outcomes derived from randomized controlled trials (RCTs) and observational studies, substantiates and provides empirical support for this premise.

In exploring the factors contributing to the limited effectiveness of pertussis vaccines against *B. parapertussis*, it is noteworthy that there is variability between *B. pertussis* and *B. parapertussis*, particularly in major protective antigens. The principal protective antigens of *B. pertussis*, namely PT, PRN, FHA, and FIM2/FIM3 fimbriae, are components of acellular pertussis vaccines [39]. *B. parapertussis*, in contrast, does not produce pertussis toxin due to a dysfunctional *ptx* operon [47]. *B. parapertussis* does not produce the fimbrial proteins FIM2 and FIM3 [48]. Polyclonal antibodies against FHA and PRN displayed weak reactivity with *B. parapertussis* proteins, and the proteins did not confer protection against *B. parapertussis* in mice models [10].

The estimated times to the last common ancestors were 0.8–4.0 million years ago (My) for *B. parapertussis* and *B. pertussis*, and both species underwent a significant loss of genes compared to their common ancestor. The number of unique genes in *B. pertussis* and *B. parapertussis* were 114 and 50, respectively [49]. Comparative analysis of the amino acid sequences of FHA, PRN, FIM2, and FIM3 revealed sequence identities of approximately 98%, 91%, 71%, and 92%, respectively, between *B. pertussis* and *B. parapertussis* [50]. Antibodies targeting the pertactin antigen are essential in establishing immunity against *B. pertussis* [51]. Isolates of *B. parapertussis* that are deficient in pertactin (PRN–) have been reported. The first PRN– *B. parapertussis* isolate was documented in 2004, and since 2007, approximately 94.3% of all collected *B. parapertussis* isolates in France have lacked PRN [52]. These variations in antigenic composition among *B. parapertussis* isolates could influence the degree of cross-protection afforded by the pertussis vaccines. The lipopolysaccharide (LPS) molecules also exhibit variations, with *B. pertussis* containing a complex trisaccharide, *B. parapertussis* featuring an altered trisaccharide, and an O-antigen-like repeat [53]. The O antigen enabled *B. parapertussis* to evade the immunity induced by the *B. pertussis* vaccine by obstructing, binding to, and affecting the functions of cross-reactive antibodies. Furthermore, O-antigen inhibited the antibody-mediated opsonization and phagocytosis induced by both aP and wP vaccines [34].

While the formulation of an independent vaccine for *B. parapertussis* remains a viable option, the integration of specific protective antigens from both *B. pertussis* and *B. parapertussis* is emerging as a strategic and efficient pathway, particularly. The lipopolysaccharide (LPS)-O antigen was identified as a critical protective antigen of *B. parapertussis* in a mouse model [54]. Studies have shown that outer membrane vesicles (OMVs) from *B. pertussis*, which incorporate the LPS-O antigen from *B. parapertussis*, effectively protect mice against infections caused by both pathogens [55]. This approach can significantly expand the effectiveness of pertussis vaccination programs, ensuring comprehensive protection against both pathogens. Another approach was elucidated by Hayes et al. (2011), who demonstrated the efficacy of the recombinant IRP1-3 antigen, which shows a high degree of conservation between *B. pertussis* and *B. parapertussis*, in eliciting a robust antibody response [56].

In conjunction with the insights derived from various studies, our systematic review contributes to the growing body of evidence that underscores the inadequacy of pertussis vaccines in conferring protection against *B. parapertussis*. The increasing incidence of *B. parapertussis* cases and outbreaks, coupled with the absence of cross-protection provided by pertussis vaccines, underscores the significance of incorporating antigens with the capacity to protect against *B. parapertussis* in both whole-cell and acellular pertussis vaccines.

## 5. Conclusions

This systematic review and meta-analysis has yielded a finding that pertussis vaccines do not exhibit a significant protective effect against *B. parapertussis* infection. While our study benefits from its systematic approach and adherence to PRISMA guidelines, it encounters limitations primarily rooted in the available literature. The paucity of recent data and a limited number of eligible studies, particularly RCTs, signifies a critical gap that warrants future research endeavors. The increasing incidence of *B. parapertussis* cases and outbreaks, combined with the lack of cross-protection conferred by pertussis vaccines, underscores the need to develop vaccine formulations that specifically target *B. parapertussis*. These findings provide valuable insights for the ongoing discussions regarding pertussis vaccination strategies, underscoring the imperative need for further research in this field to inform public health policies and practices effectively.

## Figures and Tables

**Table 1:** Characteristics of included studies.

Sr No	Author, Year	Country	Study Design	Study Period Including Follow-Up	Age at Enrolment	Types of Intervention	Types of Comparison	Number of Participants	Total Vaccinated	Vaccinated and Positive for Bpp	Vaccinated and Negative for Bpp	Total Unvaccinated	Unvaccinated and Positive for Bpp	Unvaccinated and Negative for Bpp	Reference
1	Stehr K et al., 1998	Germany	RCT	May 1991 to December 1994	2–4 months	DTP, DTaP	Unvaccinated (DT)	10,271	8532	135	8397	1739	27	1712	[12]
2	Mastrantonio P et al., 1998	Italy	RCT	September 1992 to September 1995	2 months	DTaP CB and DTaP SB, DTPw	Unvaccinated (DT)	15,601	14,040	72	13,968	1561	4	1557	[2]
3	Bergfors E et al., 1999	Sweden	RCT	November 1991 to November 1997	3 months	DTaP	Unvaccinated (DT)	3450	1724	11	1713	1726	7	1719	[29]
4	Heininger U et al., 1999	USA	RCT	May 1991 to December 1994	2–4 months	DTaP, DTPw	Unvaccinated (DT)	2187	1866	59	1807	321	13	308	[30]
5	Liese JG et al., 2003	Germany	Case-control	February 1993 to May 1995	<2 years	DTaP, DTwP	Unvaccinated	14,144	12,163	56	12,107	1981	8	1973	[31]
6	Theofiles AG et al., 2014	USA	Observational	January 2012 to December 2012	<19 years	DTP, DTaP	Unvaccinated	420	267	21	246	153	0	153	[32]
7	Muloiwa R et al., 2016	South Africa	Case-control	September 2012 to September 2013	<13 years	DTaP	Unvaccinated	460	423	10	413	37	1	36	[33]

Study details concluding author, year, study design, study period, participant age at enrollment, type of intervention and comparison, and the total number of participants, encompassing both those who were vaccinated and unvaccinated, and whether they contracted *B. parapertussis* infection or not are included RCT—randomized control trial; DTP—diphtheria pertussis tetanus toxoid containing vaccine; DTaP—diphtheria acellular pertussis tetanus toxoid containing vaccine; DTPw—diphtheria whole cellular pertussis tetanus toxoid containing vaccine; DT—diphtheria tetanus toxoid containing vaccine; SB—SmithKline Beecham; CB—Chiron Biocin.

## Data Availability

Data are available on reasonable request.

## Supplementary Materials

The following supporting information can be downloaded at: https://www.mdpi.com/article/10.3390/vaccines12030253/s1; Table S1: List of data extracted from studies included for meta-analysis; Table S2: PRISMA checklist; Table S3: Risk of bias analysis. Ref. [57] is cited in Supplementary Materials file.
